# “Mad honey and the heart: a case report of transient bradycardia and hypotension from Nepal”

**DOI:** 10.1186/s12245-026-01194-1

**Published:** 2026-03-27

**Authors:** Nabin Pahari, Mukesh Pahari, Sagun Ghimire, Prabhat Kaphle, Bharat Khatri, Rajesh Yadav, Sakshi Kumari, Mohammed Muzammil

**Affiliations:** 1 Mercy City Hospital, Rupandehi, Nepal; 2 Devdaha Medical College and Research Institute, Rupandehi, Nepal; 3 Kist Medical College and Teaching Hospital, Kathmandu, Nepal; 4 National Health Action Force, Kathmandu, Nepal; 5 B.P Koirala College of Health Sciences, Sunsari, Nepal; 6 Chitwan Medical College, Chitwan, Nepal; 7 Shimoga Institute of Medical Science, Shimoga, India; 8 Barking, Havering and Redbridge University Hospital, London, UK

**Keywords:** Bradycardia, Case report, Grayanotoxin, Honey, Nepal, Rhododendron

## Abstract

**Background:**

Consuming honey contaminated with grayanotoxins, which are derived from specific Rhododendron species (known locally as Laliguras) found in Nepal’s hilly and Himalayan regions, can result in mad honey poisoning. There are plants with high concentrations of Grayanotoxin in a number of places across the world, including Brazil, Japan, Nepal, Turkey, and North America.

**Case presentation:**

We documented a case of a 59-year-old male exhibiting acute nausea, recurrent vomiting, dizziness, hypotension (BP 60/40 mmHg), and bradycardia (42 bpm) approximately 60–80 min following the consumption of 3–4 teaspoons of locally sourced “mad honey.” There is no history of substance abuse, abnormal movement, dyspnea, or chest pain. He was awake, fully conscious, and had no other problems with his body. The electrocardiogram showed sinus bradycardia with no ischemic changes, and the cardiac enzymes were normal. Other possible causes, such as myocardial infarction, hypovolemia, sepsis, beta-blocker overdose, and organophosphate poisoning, were ruled out. A diagnosis of mad honey (grayanotoxin) poisoning was established due to the typical clinical symptoms and a history of honey consumption. The patient received intravenous fluids and atropine, resulting in quick stabilization of vital signs. He maintained stability throughout 24 hours of observation and was discharged with advice to refrain from consuming wild or unprocessed honey. This situation highlights the importance of recognizing mad honey poisoning as a reversible factor for bradycardia and hypotension.

**Conclusion:**

Although rare, mad honey poisoning can rapidly trigger bradycardia, low blood pressure, and digestive discomfort. With early recognition and timely care, including fluids and atropine, patients typically make a full recovery. Clinicians should stay alert to this possibility, particularly in areas where wild honey is traditionally consumed. Awareness is crucial in regions where wild honey is consumed.

## Learning points

Mad honey (grayanotoxin) poisoning is a significant and reversible cause of acute bradycardia and hypotension, particularly in areas where wild or unprocessed honey is commonly ingested.

A complete dietary history is required for diagnosis, as there is no routine laboratory test for grayanotoxin toxicity, and diagnosis is essentially clinical after ruling out other causes.

Grayanotoxins work by binding to voltage-gated sodium channels and raising vagal tone, resulting in sinus bradycardia, hypotension, and gastrointestinal complaints.

Supportive treatment with intravenous fluids and atropine is usually adequate to achieve quick stabilisation and complete recovery in most individuals.

Early detection of mad honey poisoning can save superfluous examinations and interventions, allowing for timely, targeted care.

## Background

Recent studies emphasize honey’s role as a healing substance with notable antibacterial, antiviral, and antifungal effects. These impacts are mainly due to its acidic pH and enzymatic function, allowing honey to prevent the proliferation of microorganisms and aid in their elimination [1].

Mad honey poisoning occurs due to ingesting honey tainted with grayanotoxin, a neurotoxin sourced from specific Rhododendron species known as Laliguras in local language in the hilly and Himalayan areas of Nepal. The world’s largest honey bee species, the Himalayan giant honey bee (Apis laboriosa), can reach lengths of up to 3.0 cm. This species produces mad honey in the high-altitude areas of Nepal by gathering nectar from Rhododendron species that contain grayanotoxins [2].

Various areas globally, including Turkey (Black Sea region), Japan, Nepal(Himalayan and Hilly regions), North America, and Brazil, contain plants with significant amounts of grayanotoxins, potentially causing honey poisoning [3, 4]. The first recorded instances of extensive mad honey poisoning are traced to 401 BC, as detailed by the military leader Xenophon in his writings, Anabasis. Historically, mad honey has served as an aphrodisiac and a natural treatment for digestive issues like peptic ulcers, gastritis, and dyspepsia, along with intestinal disorders, arthritis, skin ailments, colds, and high blood pressure [5].

Buratti et al. found that mad honey exhibits the highest antioxidant activity compared to other honey varieties [6–10].

## Case presentation

A 59-year-old male arrived at the emergency room with chief complaints of nausea, several instances of vomiting, and feelings of dizziness. He stated that these symptoms appeared suddenly around 60–80 minutes after he ingested 3–4 teaspoons (roughly 15–20 ml) of locally sourced “mad honey”. He refused any loss of awareness, convulsions, diarrhea, chest discomfort, or trouble breathing. During the presentation, he was attentive and completely aware of the time, location, and person. He had a known diagnosis of COPD for which he used one rota inhaler of Salmeterol and fluticasone and one rota inhaler of Tiotropium bromide daily. He had no documented history of heart disease and was not using any heart-related medications. There is no record of substance use, no unusual movement, shortness of breath, or chest discomfort. Upon examination, the patient looked uneasy and frail but stayed compliant. His blood pressure measured 60/40 mmHg, and his heart rate was 42 beats per minute. He was breathing easily at 17 breaths per minute, exhibiting a normal temperature and an oxygen saturation of 95% while on room air. Cardiovascular assessment showed significant bradycardia with no unusual heart sounds present. Auscultation showed clear lungs, the abdomen was non-tender and soft, and the neurological exam indicated no deficits. The diagnosis for this patient was primarily based on the distinct history of consuming mad honey, which was soon followed by symptoms including nausea, vomiting, dizziness, hypotension, and bradycardia. The short interval between eating and the emergence of symptoms strongly indicated grayanotoxin poisoning.

**Fig. 1:**
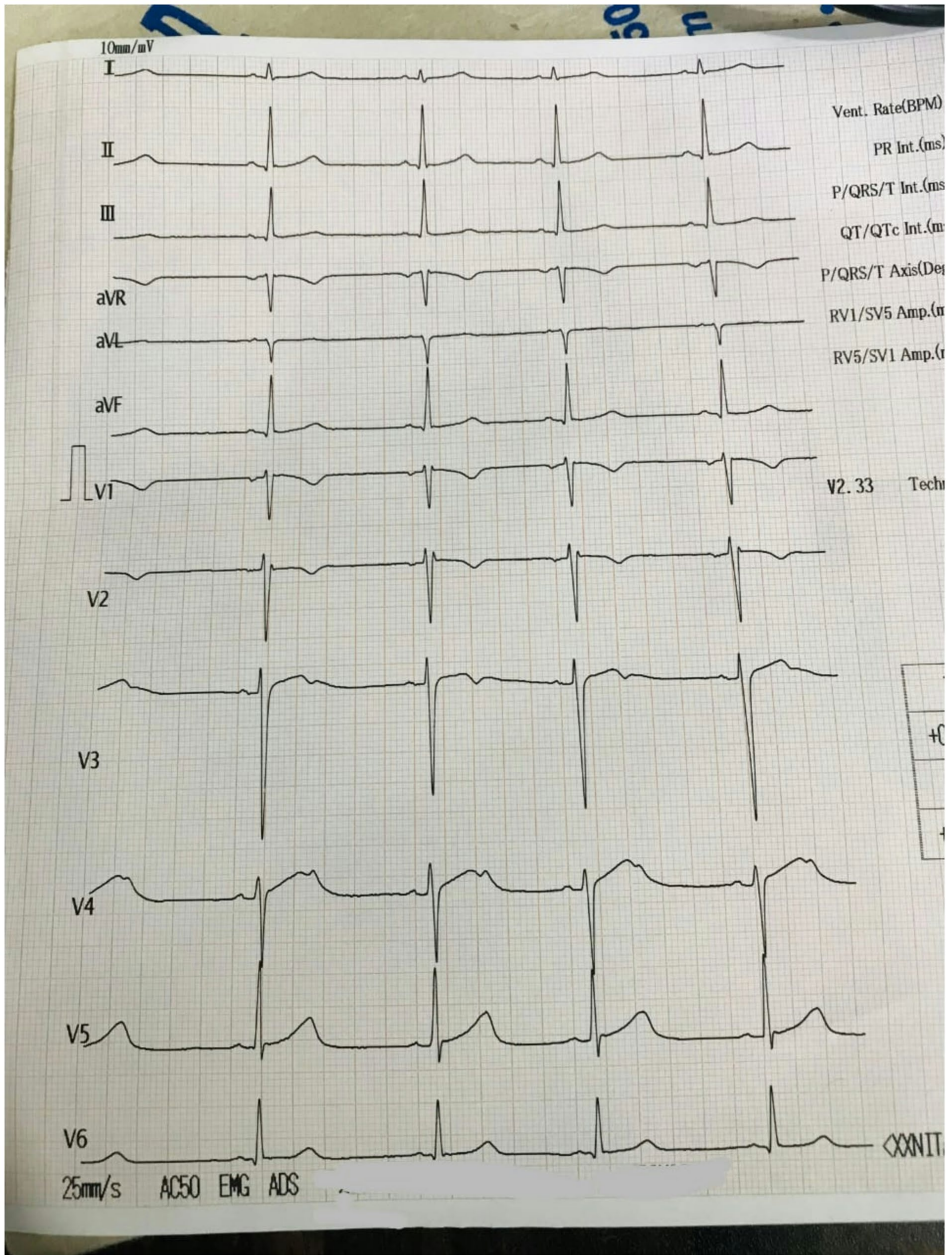
Baseline ECG at presentation showing sinus bradycardia (Heart rate 53 bpm) secondary to grayanotoxin ingestion

**Fig. 2:**
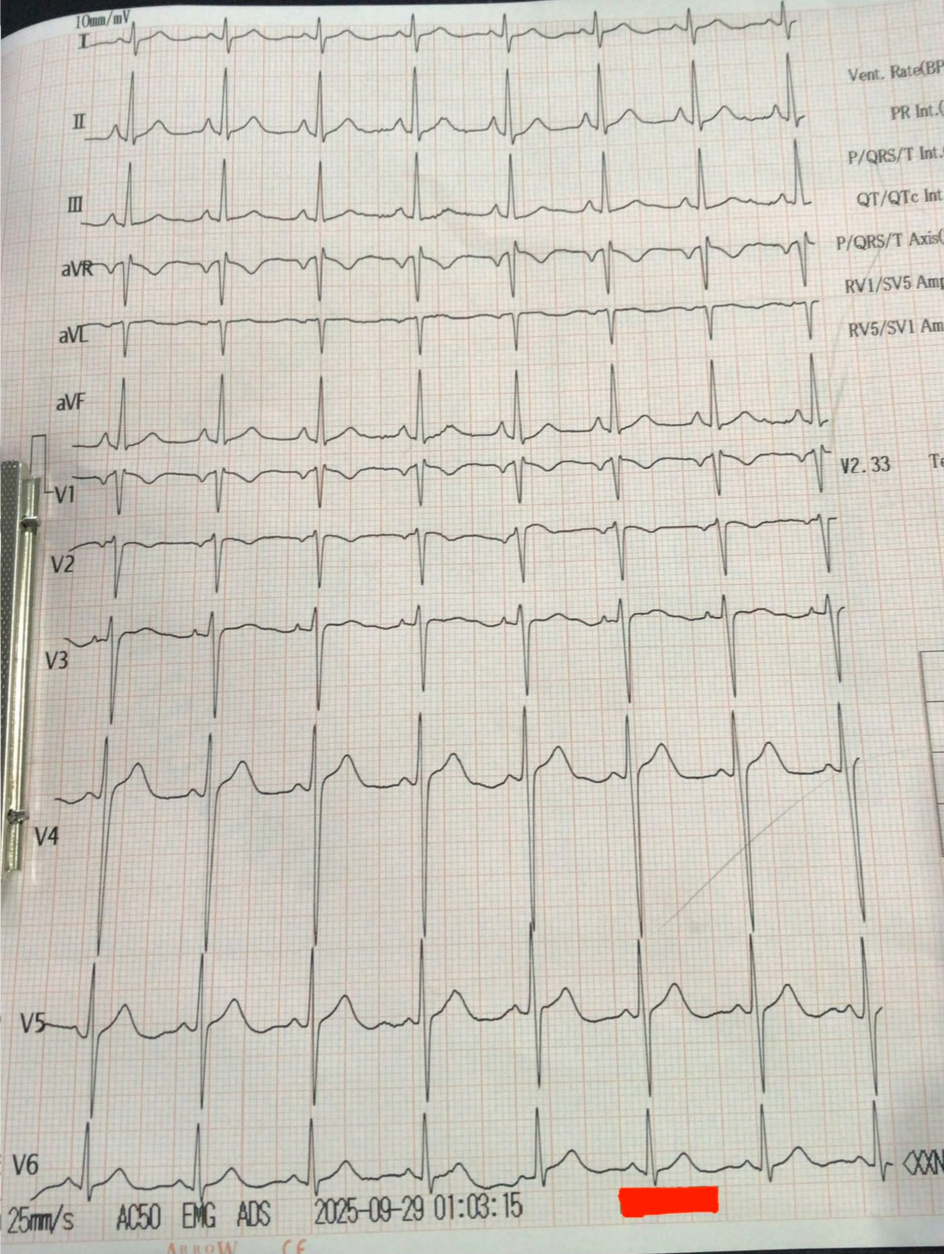
ECG revealing re-establishment of normal sinus rhythm after correction of bradycardia with Atropine

Due to the absence of a definitive laboratory test for mad honey intoxication, the diagnosis depended on thorough clinical assessment and ruling out other alternatives. We examined various differentials. Acute myocardial infarction was improbable since the patient experienced no chest pain, his cardiac enzymes were within normal limits, and the ECG displayed only sinus bradycardia without any ischemic alterations (Fig. 1). Hypovolemia and sepsis were excluded due to the lack of fluid loss, fever, or infection indicators. A beta-blocker overdose was ruled out as the patient was not taking any such medication, and organophosphate poisoning was considered improbable due to the absence of cholinergic symptoms like excessive salivation, lacrimation, urination, diarrhea, gastrointestinal cramping, vomiting, miosis, bronchorrhea, or bronchospasm. Even though grayanotoxin levels in blood can be measured, there was no any laboratory facility in our hospital or in many hospitals across Nepal. Ultimately, the reliable history of honey consumption, the distinctive clinical presentation, and the thorough elimination of other potential causes resulted in the definitive diagnosis of mad honey (grayanotoxin) poisoning. The patient received immediate treatment with intravenous fluids and a one-time administration of 0.6 mg intravenous atropine. His heart rate and blood pressure progressively improved after treatment, and no further doses of atropine was required. His ECG after atropine is shown in Fig. 2. He was monitored closely for 24 h, during which his symptoms disappeared completely and his vital signs became stable. His blood investigations are given in Table 1. His bedside transthoracic echocardiogram showed no structural abnormalities and normal wall motion. The patient was discharged the next day in stable condition and was advised to refrain from consuming wild or unprocessed honey again. He was requested to follow up after 2 weeks post-discharge. On follow up his basic blood tests and ECG results were normal.

**Table 1:** Findings from laboratory and diagnostic assessments

Test Parameters	Patient’s value	Normal Range
Hemoglobin	15 g/dl	13–17 g/dl
White blood cells	7200/ µL	4000–10,000/ µL
Platelets	250,000/ µL	150,000–450,000/ µL
Soduim	138mmol/L	135–145 mmol/L
Potassium	4.5 mmol/L	3.5-5.0 mmol/L
Urea	35 mg/dL	10–45 mg/dL
Creatinine	0.9 mg/dL	0.6–1.3 mg/dL
Glucose (Random Blood Glucose)	90 mg/dL	70–110 mg/dL
AST	26U/L	
ALT	29 U/L	
Troponin I	< 0.002 ng/mL	< 0.04 ng/mL
Total Biilirubin	0.8 mg/dL	0.3–1.2 mg/dL
Direct Biilirubin	0.2 mg/dl	0-0.3 mg/dl
Indirect Bilirubin	0.6 mg/dl	0.2-1.0 mg/dl

## Discussion

Mad honey poisoning happens when honey contaminated with grayanotoxins is ingested, which are toxins present in the flowers and leaves of plants belonging to the Ericaceae family, including the genera Rhododendron, Pieris, and Agarista.

Mad honey has historically been utilized in alternative medicine for ailments like hypertension, diabetes, and dyspepsia, though scientific proof backing these advantages is scarce. Moreover, its application as an aphrodisiac and for recreational activities is increasing [11].

Such behaviors heighten the likelihood of unintentional grayanotoxin poisoning, underscoring the necessity for healthcare professionals to be aware of the possible toxic consequences of honey consumption, particularly in areas where it is frequently eaten [12]. Grayanotoxin I is regarded as the main toxic isomer that accounts for most clinical symptoms [13]. Grayanotoxins produce their toxic effects by attaching to voltage-gated sodium channels in neuronal and cardiac cells, hindering channel inactivation and resulting in extended depolarization. Animal research suggests that grayanotoxin primarily impacts the central nervous system, leading to bradycardia and respiratory depression. Moreover, the activation of the vagus nerve via M2 muscarinic receptors also aids in reducing the heart rate [14, 15].

Grayanotoxin’s capacity to attach to the group II receptor site in voltage-gated sodium channels (Nav1•x) inside the cell is what makes it poisonous. Generally speaking, the sodium channel alpha subunit protein is made up of 24 linked membrane-spanning alpha-helixes arranged into four repeats of six alpha-helixes each (S1-S6). The pore region is aligned by the four S6 domains in the resultant channel. Desacyl asebotoxin VII, a hydrophilic grayanotoxin analog, was found to be active only when applied from the cytoplasmic side in tests using a squid axonal membrane, indicating that the binding site for granayotoxin most likely resides on the internal surface of the membrane [16]. 

Maejima et al. [17] showed that all four S6 domains play a significant role in grayanotoxin interaction in a mutation investigation of Nav1.4. By preventing sodium channel inactivation, grayanotoxin binding alters the channel’s conformation to the point where the cell is depolarized and thereby activated. The modified sodium channel’s activation potential is thus altered in the direction of hyperpolarization when grayanotoxins bind to the channel solely in its open state [17]. It’s unclear whether grayanotoxins interact with other sodium channel family members.

Clinically, intoxication typically presents with bradycardia, hypotension, dizziness, nausea, vomiting, sweating, and syncope. Severe cases may include arrhythmias or seizures.

Mad honey toxicity results in bradycardia and sustained hypotension, primarily due to the suppression of central vasomotor centers. Activation of the unmyelinated afferent fibers of the vagus nerve lowers sympathetic output and diminishes peripheral vascular resistance. This mechanism results in cholinergic-like symptoms, typical of grayanotoxin poisoning [18].

Intoxication occurs after consuming approximately 15–30 g of mad honey, with symptoms manifesting around 2.8 ± 1.8 h post-ingestion [1, 19].

## Conclusion

Mad honey poisoning, though rare, can lead to abrupt bradycardia, hypotension, and gastrointestinal issues, especially in areas where wild or unrefined honey is regularly consumed. Due to the absence of a definitive laboratory test for grayanotoxin exposure, diagnosis primarily relies on a thorough dietary history and the identification of characteristic clinical signs. Supportive treatment, such as IV fluids and atropine for symptomatic bradycardia, is frequently beneficial, and the majority of patients make a full recovery. Clinicians need to recognize the potential for mad honey poisoning in patients with unexplained bradyarrhythmias and hypotension to facilitate prompt diagnosis and appropriate treatment. A survey of the literature revealed no confirmed deaths caused by grayanotoxin (mad honey) intoxication and published case series consistently show a benign, reversible course with supportive treatment.

## Data Availability

No datasets were generated or analysed during the current study.

## Abbreviations

GCS: Glasgow coma scale
ECG: Electrocardiogram
Bpm: Beats per minute
COPD: Chronic Obstructive Pulmonary Disease
